# Inheritance as a Cause of Drunkenness

**Published:** 1883-10

**Authors:** J. G. Jewell


### Inheritance as a Cause of Drunkenness.

There is no doubt that *inheritance* has much to do with a thirst for strong drink, especially if we can bring the case down to a fine point, and find the child was begotten during the time either parent was suffering from alcoholism. As an example of this theory, I have a well authenticated case of inherited inebriety now under treatment at the "Home"—a gentleman, the third son of his parents, who is sorely afflicted with alcoholism. He tells me that himself and younger brother (the fourth son) have always, almost from infancy, been too fond of liquors, while his two elder brothers are strong total abstinence men, and never touch liquor; they are also men of wealth, while the younger, who are inebriates, are poor. He tells me that he has often heard his mother say of his father, that during the first five years of their married life, he (the father) did not use liquor in any way, and would not associate with men who did. But about the fifth year after their marriage, about the time the third son was begotten, the father had many business reverses, took to drink, and died after being an habitual drunkard for several years.—*Report of Dr. J. G. Jewell to California State Medical Society.*

